# Crystal structures and Hirshfeld surface analysis of *trans*-bis­(thio­cyanato-κ*N*)bis­{2,4,6-trimethyl-*N*-[(pyridin-2-yl)methyl­idene]aniline-κ^2^ *N*,*N*′}manganese(II) and *trans*-bis­(thio­cyanato-κ*N*)bis­{2,4,6-trimethyl-*N*-[(pyridin-2-yl)methyl­idene]aniline-*κ* ^2^ *N*,*N*′}nickel(II))

**DOI:** 10.1107/S2056989020000870

**Published:** 2020-01-31

**Authors:** Siripak Jittirattanakun, Chatphorn Theppitak, Nanthawat Wannarit, Bachari Rotsut, Kittipong Chainok

**Affiliations:** a Division of Chemistry, Faculty of Science and Technology, Thammasat University, Khlong Luang, Pathum Thani, 12121, Thailand; b Wisetchaichan Tantiwittayapoom School, Wisetchaichan, Angthong, 14110, Thailand; c Materials and Textile Technology, Faculty of Science and Technology, Thammasat University, Khlong Luang, Pathum Thani, 12121, Thailand

**Keywords:** crystal structure, manganese(II), nickel(II), Schiff base, weak inter­molecular inter­actions, Hirshfeld surface analysis

## Abstract

Two new mononuclear metal complexes involving the bidentate Schiff base ligand 2,4,6-trimethyl-*N*-[(pyridin-2-yl)methyl­idene]aniline (C_15_H_16_N_2_ or PM-TMA), [Mn(NCS)_2_(PM-TMA)_2_] (**I**) and [Ni(NCS)_2_(PM-TMA)_2_] (**II**), were synthesized and their structures determined by single-crystal X-ray diffraction. Although the title compounds crystallize in different crystal systems [triclinic for (**I**) and monoclinic for (**II**)], both asymmetric units consist of one-half of the complex mol­ecule, *i.e.* one metal(II) cation, one PM-TMA ligand, and one N-bound thio­cyanate anion. In both complexes, the metal(II) cation is located on a centre of inversion and adopts a distorted octa­hedral coordination environment defined by four N atoms from two symmetry-related PM-TMA ligands in the equatorial plane and two N atoms from two symmetry-related NCS^−^ anions in a *trans* axial arrangement. The tri­methyl­benzene and pyridine rings of the PM-TMA ligand are oriented at dihedral angles of 74.18 (7) and 77.70 (12)° for (**I**) and (**II**), respectively. The subtle change in size of the central metal cations leads to a different crystal packing arrangement for (**I**) and (**II**) that is dominated by weak C—H⋯S, C—H⋯π, and π–π inter­actions. Hirshfeld surface analysis and two-dimensional fingerprint plots were used to qu­antify these inter­molecular contacts, and indicate that the most significant contacts in packing are H⋯H [48.1% for (**I**) and 54.9% for (**II**)], followed by H⋯C/C⋯H [24.1% for (**I**) and 15.7% for (**II**)], and H⋯S/S⋯H [21.1% for (**I**) and 21.1% for (**II**)].

## Abstract 2

Each metal cation in the mol­ecular structures of [Mn(NCS)_2_(PM-TMA)_2_] (**I**) and [Ni(NCS)_2_(PM-TMA)_2_] (**II**) (PM-TMA is 2,4,6-trimethyl-*N*-[(pyridin-2-yl)methyl­idene]aniline) is situated on an inversion centre and is in a distorted octa­hedral coordination by six N atoms.

## Chemical context   

Schiff bases are widely employed as ligands in the development of coordination chemistry (Liu & Hamon, 2019 ▸). Among them, derivatives of the Schiff base 2-pyridyl­methanimine have been used as chelating ligands in the construction of discrete metal complexes exhibiting inter­esting luminescent properties (Basu Baul *et al.*, 2013 ▸), magnetic spin-crossover behaviour (Létard *et al.*, 1997 ▸; Capes *et al.*, 2000 ▸), or biological and catalytic reactivities (Cozzi, 2004 ▸; Creaven *et al.*, 2010 ▸). These ligands are also able to generate non-covalent inter­actions such as hydrogen bonding and π–π stacking that aid in stabilizing the assembly and provide diversity to the architectures of the crystal structures. On the other hand, pseudohalides such as thio­cyanate (NCS^−^) and seleno­cyanate (NCSe^−^) anions are a class of rigid ligands with either a terminal or a bridging coordination behaviour. They have been employed extensively with neutral *N*-donor co-ligands in the development of novel functional coordination materials, particularly in the field of mol­ecular-based magnets (Suckert *et al.*, 2016 ▸).
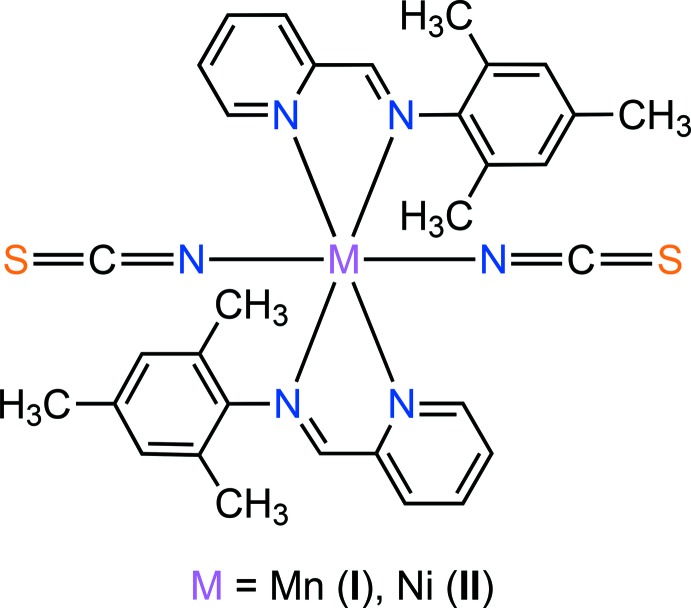


In this work, we combined 2-pyridine­carboxaldehyde and 2,4,6-tri­methyl­aniline to synthesize a new bidentate Schiff base ligand with two potential *N*,*N*′-donor sites, *viz*. 2,4,6-trimethyl-*N*-[(pyridin-2-yl)methyl­idene]aniline (C_15_H_16_N_2_ or PM-TMA). Reaction of the PM-TMA ligand and *M*(NCS)_2_ precursors (*M* = Mn, Ni) in methano­lic solutions resulted in the formation of discrete mononuclear complexes with the formula [*M*(NCS)_2_(PM-TMA)_2_], *M* = Mn (**I**), Ni (**II**). Their mol­ecular and crystal structures as well as Hirshfeld surface analysis are reported.

## Structural commentary   

**Figure 1:**
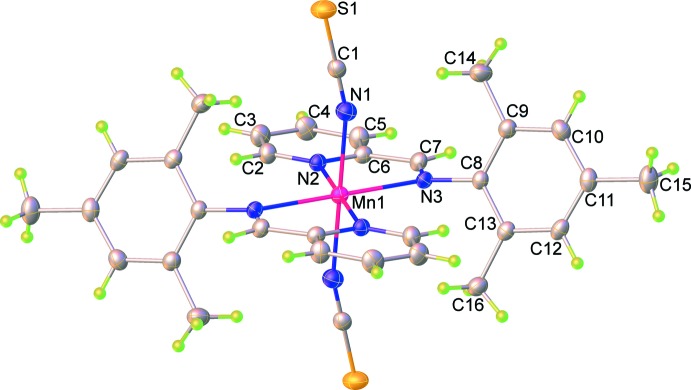
Mol­ecular structures of the title complex (**I**), showing the atom-labelling scheme. Displacement ellipsoids are drawn at the 50% probability level.

**Figure 3:**
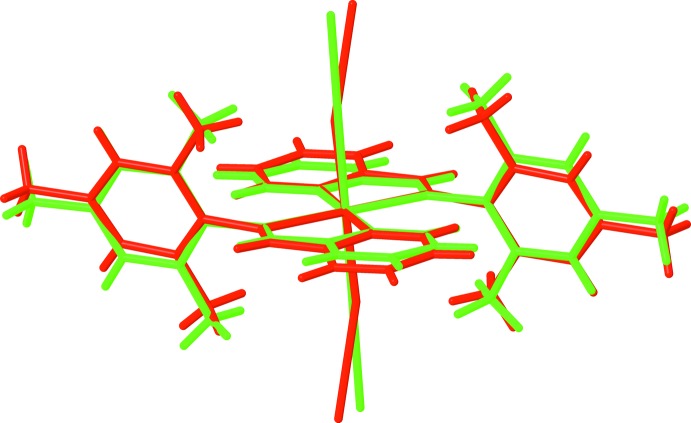
View of the structure overlay of (**I**) (red) and (**II**) (green).

The mol­ecular structures of (**I**) and (**II**) are shown in Fig. 1 ▸ and 2 ▸. Although the title compounds crystallize in different space groups [*P* 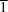 for (**I**) and *P*2_1_/*n* for (**II**)], in both cases the asymmetric unit consist of one-half of the mol­ecule, *i.e.* one metal(II) cation, one PM-TMA ligand and one thio­cyanate anion. Each metal(II) cation is located on a centre of inversion and adopts a distorted octa­hedral coordination environment with four N atoms from two symmetry-related PM-TMA ligands in the equatorial plane and two N atoms from symmetry-related NCS^−^ anions in a *trans* axial arrangement. The *M*—N bond lengths [2.174 (2) to 2.312 (2) Å for (**I**) and 2.027 (3) to 2.184 (2) Å for (**II**)] and N—*M*—N bond angles [74.27 (6) to 180° for (**I**) and 78.4 (1) to 180° for (**II**)] for both complexes are all in the normal range for similar Schiff base complexes with Mn^II^ (Chattopadhyay *et al.*, 2002 ▸; Lucas *et al.*, 2005 ▸) and Ni^II^ (Guo, 2009 ▸; Layek *et al.*, 2014 ▸) ions. Note that the Mn1—N1—C1 bond angle [164.3 (2)°] in (**I**) is somewhat more bent than the corresponding Ni1—N1—C1 bond angle [176.7 (3)°] in (**II**). The PM-TMA ligands are not co-planar, with the tri­methyl­benzene ring oriented to the pyridine ring at a dihedral angle of 74.18 (7) and 77.70 (12)° for (**I**) and (**II**), respectively. An overlay of the complex mol­ecules of (**I**) and (**II**) is illustrated in Fig. 3 ▸, showing the slight differences in orientation of the tri­methyl­benzene rings and thio­cyanate anions. This impacts significantly upon the mol­ecular packing as described in the next section.

## Supra­molecular features   

**Figure 4:**
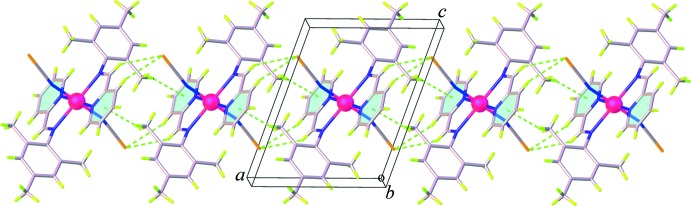
View of chains in (**I**) generated through C—H⋯S and C—H⋯π inter­actions.

**Figure 5:**
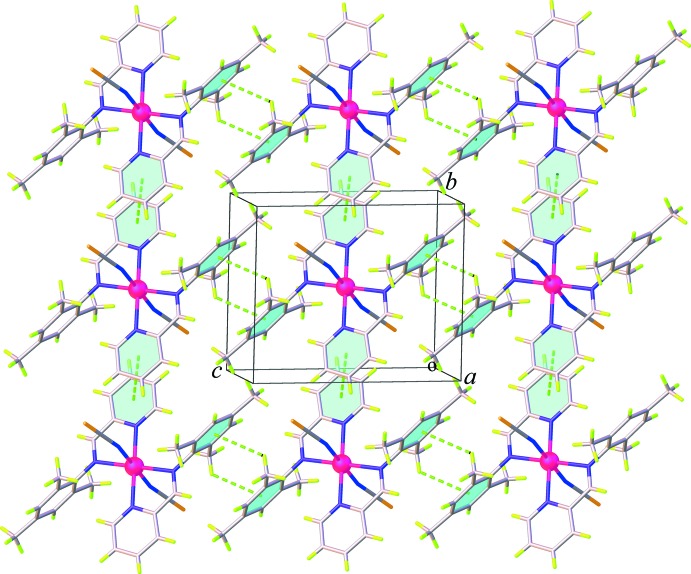
View of the three-dimensional network in (**I**) generated through C—H⋯π and π–π inter­actions.

**Figure 6:**
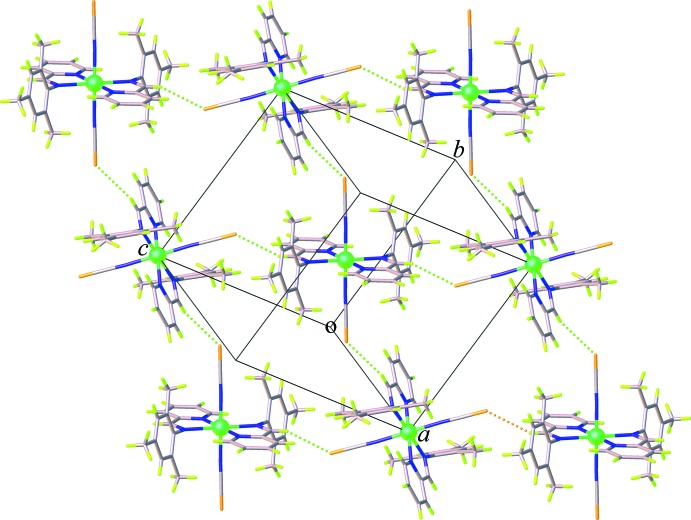
Formation of a two-dimensional supra­molecular network in (**II**) generated through C—H⋯S hydrogen bonds.

**Figure 7:**
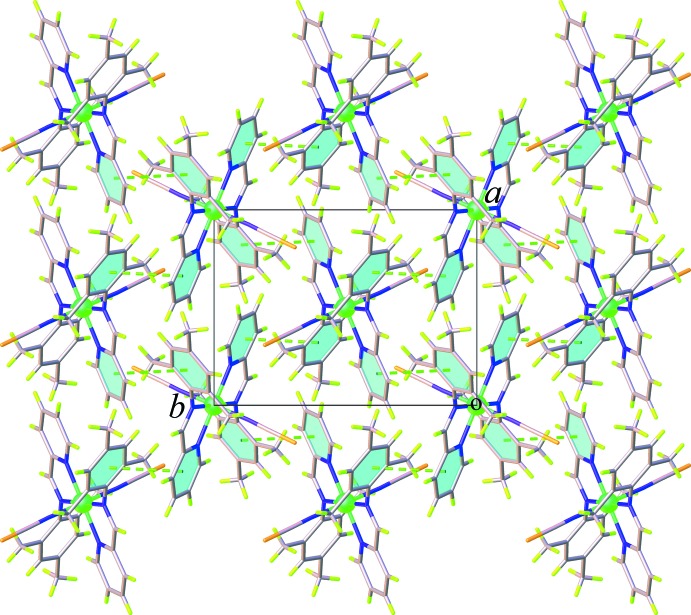
Crystal packing of (**II**) viewed along the *c* axis with aromatic π–π stacking inter­actions bonds shown as dashed lines.

The crystal packing of (**I**) and (**II**) is dominated by weak C—H⋯S, C—H⋯π, and π–π inter­actions. In the crystal structure of (**I**), pairs of weak C—H⋯S hydrogen bonds along with the C—H⋯π inter­actions involving the methyl H atoms (H14*A* and H14*B*) and the tri­methyl­benzene ring or the midpoint of thio­cyanate C=N group, Table 1 ▸, link inversion-related mol­ecules into a chain running parallel to the *a* axis, Fig. 4 ▸. The chains are further linked into a three-dimensional supra­molecular network through weak π–π inter­actions involving the pyridine rings [centroid-to-centroid distance = 3.909 (3) Å] and additional weak C—H⋯π inter­actions between the methyl H atoms and the tri­methyl­benzene rings, Fig. 5 ▸ (Table 1 ▸). For (**II**), adjacent mol­ecules are linked tog­ether into a sheet extending parallel to (101), Fig. 6 ▸, by weak C—H⋯S hydrogen bonds between the methine C—H groups and the thio­cyanate S atoms, Table 2 ▸. On the other hand, as seen in Fig. 7 ▸, the packing in (**II**) also features weak π–π stacking inter­actions arising from the pyridine rings and the tri­methyl­benzene rings [centroid-to-centroid distance = 4.147 (3) Å, dihedral angle = 17.41 (14)°]. There is an additional C—H⋯π inter­action between the methyl H atom and the midpoint of the thio­cyanate C=N group (Table 2 ▸). These inter­actions help to enhance the dimensionality into a three-dimensional supra­molecular architecture.

## Hirshfeld surface analysis   

**Figure 8:**
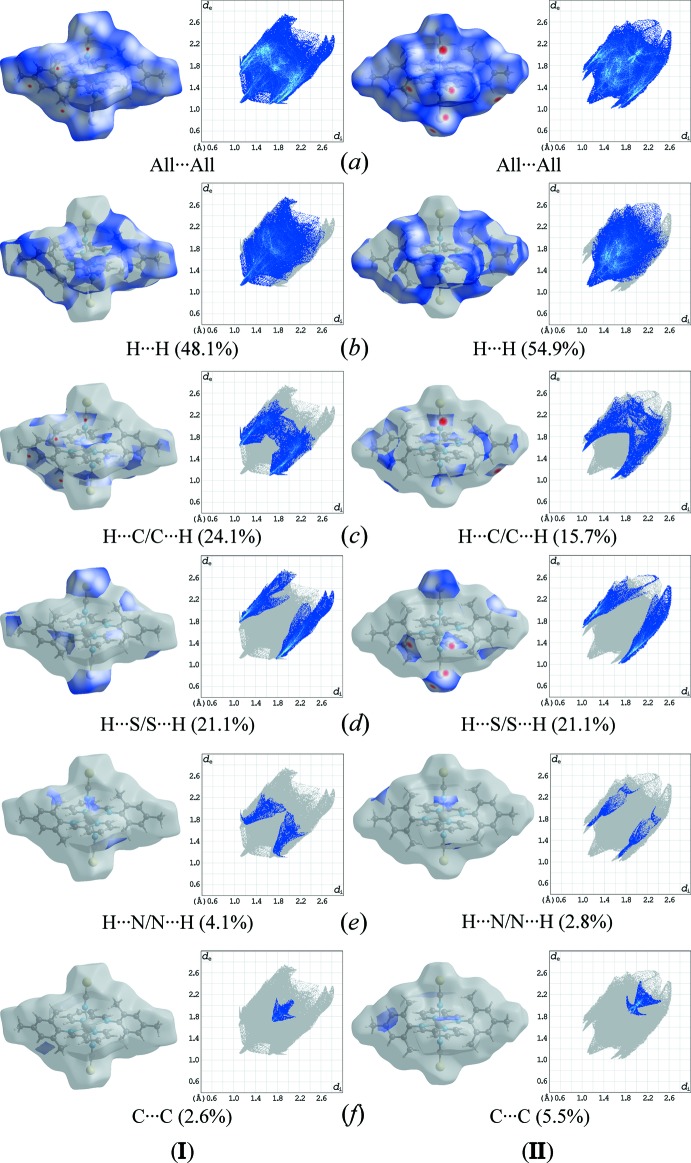
Two-dimensional fingerprint plots of (**I**) and (**II**), showing (*a*) all inter­actions, and those delineated into (*b*) H⋯H, (*c*) H⋯C/C⋯H, (*d*) H⋯S/S⋯H, (*e*) H⋯N/N⋯H, and (*f*) C⋯C contacts [*d*_e_ and d_i_ represent the distances from a point on the Hirshfeld surface to the nearest atoms outside (external) and inside (inter­nal) the surface, respectively].

The inter­molecular inter­actions between the mol­ecules in the crystal structures of (**I**) and (**II)** were qu­anti­fied by Hirshfeld surface analysis (McKinnon *et al.*, 2007 ▸) and two-dimensional fingerprint plots (Spackman & McKinnon, 2002 ▸) generated by *CrystalExplorer* (Turner *et al.*, 2017 ▸); results are shown in Figs. 8 ▸ and 9 ▸, respectively. Major contributions to the *d*_norm_ surfaces in both cases are H⋯H contacts [48.1% for (**I**) and 54.9% for (**II**)], which represent van der Waals inter­actions. Minor contributions are due to H⋯C/C⋯H [24.1% for (**I**) and 15.7% for (**II**)] and H⋯S/S⋯H (21.1% for (**I**) and 21.1% for (**II**)) contacts, associated with weak C—H⋯π and C—H⋯S inter­actions, respectively. These contributions are characterized as bright-red spots on the Hirshfeld surface mapped over *d*_norm_ and are observed as two sharp peaks in the two-dimensional plots. The C⋯C contacts associated with aromatic π—π stacking contribute only with a small percentage in (**I**) (2.6%) and about twice the amount in (**II**) (5.5%). H⋯N/N⋯H contacts in both cases are negligible.

## Database survey   

A search of the Cambridge Structural Database (CSD, Version 5.40, last update August 2019; Groom *et al.*, 2016 ▸) revealed no match for coordination compounds with the Schiff base 2,4,6-trimethyl-*N*-[(pyridin-2-yl)methyl­idene]aniline. A general search for thio­cyanato coordination compounds involving transition metals and *N*-[(pyridin-2-yl)methyl­idene]aniline as the main skeleton resulted in 122 structures with different substituents on the benzyl rings. In these structures, the two bidentate Schiff base ligands and the two thio­cyanate anions are octa­hedrally arranged around the central metal cations in a *cis*-conformation. There is only one complex with a *trans*-conformation and the same skeleton as in the title complexes, *viz. trans*-[Cd(NCS)_2_(C_14_H_14_N_2_)_2_] (CSD refcode GARTAW; Malekshahian *et al.*, 2012 ▸). In this complex, weak C—H⋯S hydrogen bonds consolidate the crystal packing, similar to the title complexes (**I**) and (**II**).

## Synthesis and crystallization   

All reagents were of analytical grade and were used as received without further purification. The bidentate Schiff base ligand, 2,4,6-trimethyl-*N*-[(pyridin-2-yl)methyl­idene]aniline (C_15_H_16_N_2_ or PM-TMA) was synthesized according to a literature method (Theppitak *et al.*, 2014 ▸). A solution of PM-TMA (89.7 mg, 0.4 mmol) in methanol (5 ml) was placed in a test tube. To a solution of Mn(ClO_4_)_2_·6H_2_O (50.8 mg, 0.2 mmol) in methanol (5 ml) was added KNCS (39.0 mg, 0.4 mmol), and the solution was stirred at room temperature for 30 min and then filtered to remove a white precipitate of KClO_4_. The solution was then carefully layered on the methanol solution of PM-TMA. After slow diffusion at room temperature for 3 d, yellow block-shaped crystals of (**I**) were obtained in 88% yield (44.7 mg) based on the manganese(II) source. Complex (**II**) was prepared following the procedure described above for (**I**), except that Ni(ClO_4_)_2_·6H_2_O (58.2 mg, 0.2 mmol) and KNCS (39.0 mg, 0.4 mmol) were used. Yellow block-shaped crystal of (**II**) were obtained in 82% yield (47.7 mg) based on the nickel(II) source.

## Refinement   

Crystal data, data collection and structure refinement details are summarized in Table 3 ▸. All C-bound H atoms were placed in calculated positions and refined using a riding model with C—H = 0.93–0.97 Å and with *U*_iso_(H) = 1.2–1.5*U*_eq_(C).

## Supplementary Material

Crystal structure: contains datablock(s) 1, 2. DOI: 10.1107/S2056989020000870/wm5536sup1.cif

Structure factors: contains datablock(s) 1. DOI: 10.1107/S2056989020000870/wm55361sup2.hkl

Supporting information file. DOI: 10.1107/S2056989020000870/wm55361sup4.cdx

Structure factors: contains datablock(s) 2. DOI: 10.1107/S2056989020000870/wm55362sup3.hkl

CCDC references: 1979499, 1979498

Additional supporting information:  crystallographic information; 3D view; checkCIF report

## Figures and Tables

**Figure 2:**
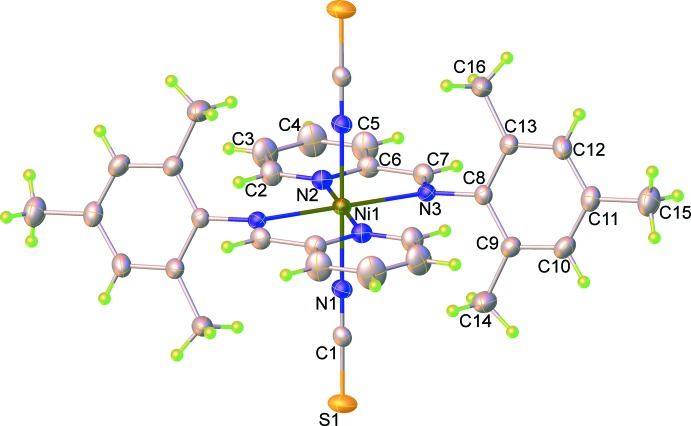
Mol­ecular structures of the title complex (**II**), showing the atom-labelling scheme. Displacement ellipsoids are drawn at the 50% probability level.

**Figure 9:**
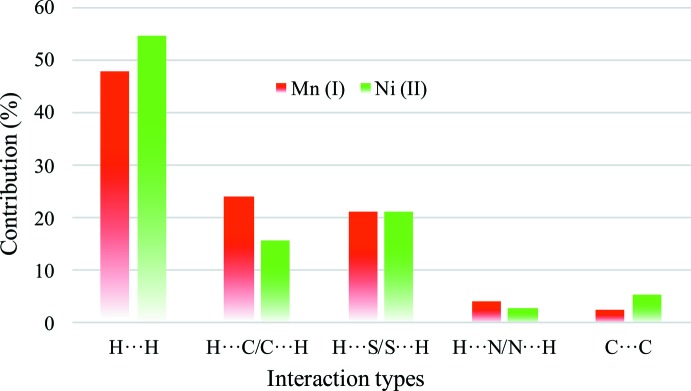
Qu­anti­tative results of different inter­molecular contacts contributing to the Hirshfeld surfaces of (**I**) and (**II**).

**Table 1:** Hydrogen-bond geometry (Å, °) for (**I**) *Cg*1 is the mid-point of the C1=N1 group. *Cg*2 and *Cg*3 are the centroids of the N2/C2–C6 and C8–C13 rings.

*D*—H⋯*A*	*D*—H	H⋯*A*	*D*⋯*A*	*D*—H⋯*A*
C3—H3⋯S1^i^	0.93	3.16	3.812 (3)	129
C4—H4⋯*Cg*1^i^	0.93	2.89	3.561 (3)	129
C5—H5⋯S1^ii^	0.93	3.00	3.785 (3)	143
C7—H7⋯S1^ii^	0.93	3.07	3.871 (2)	146
C14—H14*A*⋯*Cg*1^ii^	0.96	2.89	3.779 (3)	153
C14—H14*B*⋯*Cg*2^ii^	0.96	2.68	3.556 (3)	152
C16—H16*C*⋯*Cg*3^iii^	0.96	3.14	3.711 (3)	145

Symmetry codes: (i) 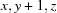; (ii) 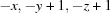; (iii) 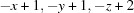.

**Table 2:** Hydrogen-bond geometry (Å, °) for (**II**) *Cg*1 is the mid-point of the C1=N1 group.

*D*—H⋯*A*	*D*—H	H⋯*A*	*D*⋯*A*	*D*—H⋯*A*
C4—H4⋯S1^i^	0.93	2.89	3.769 (4)	158
C7—H7⋯S1^ii^	0.93	2.83	3.680 (3)	153
C10—H10⋯S1^iii^	0.93	3.17	3.871 (4)	134
C10—H10⋯*Cg*1^iii^	0.93	2.73	3.655 (4)	171

Symmetry codes: (i) 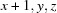; (ii) 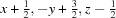; (iii) 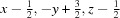.

**Table 3:** Experimental details

	(**I**)	(**II**)
Crystal data		
Chemical formula	[Mn(NCS)_2_(C_15_H_16_N_2_)_2_]	[Ni(NCS)_2_(C_15_H_16_N_2_)_2_]
*M*_r_	619.69	623.46
Crystal system, space group	Triclinic, *P* 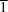	Monoclinic, *P*2_1_/*n*
Temperature (K)	296	296
*a*, *b*, *c* (Å)	8.5597 (5), 9.0255 (5), 10.7718 (7)	9.6156 (5), 12.6786 (7), 12.9870 (7)
α, β, γ (°)	91.718 (2), 109.830 (2), 95.080 (2)	90, 101.250 (2), 90
*V* (Å^3^)	778.16 (8)	1552.85 (14)
*Z*	1	2
Radiation type	Mo *K*α	Mo *K*α
μ (mm^−1^)	0.59	0.79
Crystal size (mm)	0.28 × 0.28 × 0.22	0.34 × 0.3 × 0.3

Data collection		
Diffractometer	Bruker D8 QUEST CMOS PHOTON II	Bruker D8 QUEST CMOS PHOTON II
Absorption correction	Multi-scan (*SADABS*; Krause *et al.*, 2015 ▸)	Multi-scan (*SADABS*; Krause *et al.*, 2015 ▸)
*T*_min_, *T*_max_	0.673, 0.745	0.607, 0.745
No. of measured, independent and observed [*I* > 2σ(*I*)] reflections	7594, 3091, 2326	16977, 3037, 2486
*R*_int_	0.043	0.032
(sin θ/λ)_max_ (Å^−1^)	0.625	0.618

Refinement		
*R*[*F* ^2^ > 2σ(*F* ^2^)], *wR*(*F* ^2^), *S*	0.044, 0.100, 1.02	0.053, 0.104, 1.21
No. of reflections	3091	3037
No. of parameters	190	190
H-atom treatment	H-atom parameters constrained	H-atom parameters constrained
Δρ_max_, Δρ_min_ (e Å^−3^)	0.27, −0.20	0.70, −0.47

Computer programs: *APEX3* and *SAINT* (Bruker, 2016 ▸), *SHELXT* (Sheldrick, 2015*a* ▸), *SHELXL* (Sheldrick, 2015*b* ▸) and *OLEX2* (Dolomanov *et al.*, 2009 ▸).

## supplementary crystallographic information

### trans-Bis(thiocyanato-κN)bis{2,4,6-trimethyl-N-[(pyridin-2-yl)methylidene]aniline-κ2N,N'}manganese(II) (1) Crystal data

**Table:**

[Mn(NCS)_2_(C_15_H_16_N_2_)_2_]	*Z* = 1
*M**_r_* = 619.69	*F*(000) = 323
Triclinic, *P*1	*D*_x_ = 1.322 Mg m^−^^3^
*a* = 8.5597 (5) Å	Mo *K*α radiation, λ = 0.71073 Å
*b* = 9.0255 (5) Å	Cell parameters from 2271 reflections
*c* = 10.7718 (7) Å	θ = 2.9–25.8°
α = 91.718 (2)°	µ = 0.59 mm^−^^1^
β = 109.830 (2)°	*T* = 296 K
γ = 95.080 (2)°	Block, yellow
*V* = 778.16 (8) Å^3^	0.28 × 0.28 × 0.22 mm

### trans-Bis(thiocyanato-κN)bis{2,4,6-trimethyl-N-[(pyridin-2-yl)methylidene]aniline-κ2N,N'}manganese(II) (1) Data collection

**Table:**

BRUKER D8 QUEST CMOS PHOTON II diffractometer	3091 independent reflections
Radiation source: sealed x-ray tube	2326 reflections with *I* > 2σ(*I*)
Graphite monochromator	*R*_int_ = 0.043
Detector resolution: 7.39 pixels mm^-1^	θ_max_ = 26.4°, θ_min_ = 2.9°
φ and ω scans	*h* = −10→10
Absorption correction: multi-scan (*SADABS*; Krause *et al.*, 2015)	*k* = −11→11
*T*_min_ = 0.673, *T*_max_ = 0.745	*l* = −13→13
7594 measured reflections	

### trans-Bis(thiocyanato-κN)bis{2,4,6-trimethyl-N-[(pyridin-2-yl)methylidene]aniline-κ2N,N'}manganese(II) (1) Refinement

**Table:**

Refinement on *F*^2^	0 restraints
Least-squares matrix: full	Hydrogen site location: inferred from neighbouring sites
*R*[*F*^2^ > 2σ(*F*^2^)] = 0.044	H-atom parameters constrained
*wR*(*F*^2^) = 0.100	*w* = 1/[σ^2^(*F*_o_^2^) + (0.0454*P*)^2^ + 0.1287*P*] where *P* = (*F*_o_^2^ + 2*F*_c_^2^)/3
*S* = 1.02	(Δ/σ)_max_ < 0.001
3091 reflections	Δρ_max_ = 0.27 e Å^−^^3^
190 parameters	Δρ_min_ = −0.20 e Å^−^^3^

### trans-Bis(thiocyanato-κN)bis{2,4,6-trimethyl-N-[(pyridin-2-yl)methylidene]aniline-κ2N,N'}manganese(II) (1) Special details

**Table:**

Geometry. All esds (except the esd in the dihedral angle between two l.s. planes) are estimated using the full covariance matrix. The cell esds are taken into account individually in the estimation of esds in distances, angles and torsion angles; correlations between esds in cell parameters are only used when they are defined by crystal symmetry. An approximate (isotropic) treatment of cell esds is used for estimating esds involving l.s. planes.

### trans-Bis(thiocyanato-κN)bis{2,4,6-trimethyl-N-[(pyridin-2-yl)methylidene]aniline-κ2N,N'}manganese(II) (1) Fractional atomic coordinates and isotropic or equivalent isotropic displacement parameters (Å^2^)

**Table:**

	*x*	*y*	*z*	*U*_iso_*/*U*_eq_	
Mn1	0.500000	0.500000	0.500000	0.03059 (16)	
S1	−0.00476 (8)	0.21358 (9)	0.19246 (7)	0.0556 (2)	
N1	0.2655 (2)	0.3678 (2)	0.3908 (2)	0.0432 (5)	
N2	0.3816 (2)	0.71929 (19)	0.47958 (17)	0.0305 (4)	
N3	0.4064 (2)	0.5223 (2)	0.67669 (17)	0.0295 (4)	
C1	0.1524 (3)	0.3041 (3)	0.3083 (2)	0.0352 (5)	
C2	0.3662 (3)	0.8173 (3)	0.3873 (2)	0.0394 (6)	
H2	0.403637	0.795727	0.317827	0.047*	
C3	0.2972 (3)	0.9498 (3)	0.3898 (3)	0.0460 (6)	
H3	0.286186	1.014330	0.322324	0.055*	
C4	0.2457 (3)	0.9843 (3)	0.4924 (3)	0.0486 (7)	
H4	0.200641	1.073625	0.496677	0.058*	
C5	0.2609 (3)	0.8853 (3)	0.5905 (2)	0.0431 (6)	
H5	0.226735	0.906801	0.661687	0.052*	
C6	0.3284 (3)	0.7535 (2)	0.5801 (2)	0.0325 (5)	
C7	0.3369 (3)	0.6414 (3)	0.6780 (2)	0.0340 (5)	
H7	0.290036	0.657904	0.742805	0.041*	
C8	0.3925 (3)	0.4146 (2)	0.7695 (2)	0.0301 (5)	
C9	0.2353 (3)	0.3410 (3)	0.7554 (2)	0.0364 (5)	
C10	0.2266 (3)	0.2367 (3)	0.8455 (2)	0.0415 (6)	
H10	0.123433	0.186156	0.836340	0.050*	
C11	0.3663 (3)	0.2049 (3)	0.9487 (2)	0.0403 (6)	
C12	0.5191 (3)	0.2774 (3)	0.9573 (2)	0.0375 (6)	
H12	0.614163	0.255307	1.024579	0.045*	
C13	0.5366 (3)	0.3818 (2)	0.8697 (2)	0.0328 (5)	
C14	0.0778 (3)	0.3699 (3)	0.6467 (3)	0.0531 (7)	
H14A	0.041645	0.462049	0.667966	0.080*	
H14B	−0.007793	0.289925	0.637957	0.080*	
H14C	0.098935	0.376187	0.564940	0.080*	
C15	0.3481 (4)	0.0982 (3)	1.0500 (3)	0.0573 (8)	
H15A	0.456430	0.073952	1.103645	0.086*	
H15B	0.280474	0.008835	1.005464	0.086*	
H15C	0.295925	0.144197	1.105062	0.086*	
C16	0.7065 (3)	0.4597 (3)	0.8867 (2)	0.0441 (6)	
H16A	0.733250	0.440009	0.808518	0.066*	
H16B	0.788622	0.423560	0.961442	0.066*	
H16C	0.705757	0.565104	0.900840	0.066*	

### trans-Bis(thiocyanato-κN)bis{2,4,6-trimethyl-N-[(pyridin-2-yl)methylidene]aniline-κ2N,N'}manganese(II) (1) Atomic displacement parameters (Å^2^)

**Table:**

	*U*^11^	*U*^22^	*U*^33^	*U*^12^	*U*^13^	*U*^23^
Mn1	0.0319 (3)	0.0273 (3)	0.0335 (3)	0.0076 (2)	0.0113 (2)	0.0037 (2)
S1	0.0379 (4)	0.0659 (5)	0.0543 (4)	−0.0008 (3)	0.0070 (3)	−0.0082 (4)
N1	0.0384 (11)	0.0424 (13)	0.0473 (12)	0.0020 (10)	0.0131 (10)	0.0040 (10)
N2	0.0318 (10)	0.0278 (10)	0.0316 (10)	0.0050 (8)	0.0096 (8)	0.0048 (8)
N3	0.0300 (9)	0.0285 (10)	0.0294 (10)	0.0065 (8)	0.0084 (8)	0.0067 (8)
C1	0.0328 (12)	0.0352 (13)	0.0415 (14)	0.0094 (11)	0.0161 (11)	0.0071 (11)
C2	0.0447 (14)	0.0362 (14)	0.0399 (14)	0.0049 (11)	0.0170 (11)	0.0080 (11)
C3	0.0515 (15)	0.0363 (14)	0.0521 (16)	0.0106 (12)	0.0177 (13)	0.0171 (12)
C4	0.0556 (16)	0.0325 (14)	0.0646 (18)	0.0183 (12)	0.0251 (14)	0.0150 (12)
C5	0.0514 (15)	0.0372 (14)	0.0469 (15)	0.0149 (12)	0.0223 (12)	0.0058 (11)
C6	0.0329 (12)	0.0285 (12)	0.0348 (13)	0.0062 (10)	0.0091 (10)	0.0043 (10)
C7	0.0373 (12)	0.0367 (14)	0.0290 (12)	0.0084 (11)	0.0115 (10)	0.0022 (10)
C8	0.0360 (12)	0.0274 (12)	0.0291 (12)	0.0082 (10)	0.0126 (10)	0.0046 (9)
C9	0.0372 (12)	0.0377 (14)	0.0358 (13)	0.0097 (11)	0.0129 (10)	0.0060 (10)
C10	0.0425 (14)	0.0372 (14)	0.0498 (15)	0.0043 (11)	0.0220 (12)	0.0078 (12)
C11	0.0583 (16)	0.0299 (13)	0.0399 (14)	0.0116 (12)	0.0240 (12)	0.0072 (10)
C12	0.0465 (14)	0.0355 (13)	0.0299 (12)	0.0156 (12)	0.0095 (11)	0.0063 (10)
C13	0.0390 (12)	0.0299 (12)	0.0288 (12)	0.0088 (10)	0.0096 (10)	0.0015 (9)
C14	0.0353 (13)	0.0594 (18)	0.0598 (18)	0.0039 (13)	0.0092 (13)	0.0166 (14)
C15	0.078 (2)	0.0481 (17)	0.0577 (18)	0.0182 (15)	0.0347 (16)	0.0215 (14)
C16	0.0401 (13)	0.0474 (16)	0.0401 (14)	0.0053 (12)	0.0071 (11)	0.0077 (12)

### trans-Bis(thiocyanato-κN)bis{2,4,6-trimethyl-N-[(pyridin-2-yl)methylidene]aniline-κ2N,N'}manganese(II) (1) Geometric parameters (Å, º)

**Table:**

Mn1—N1^i^	2.174 (2)	C7—H7	0.9300
Mn1—N1	2.174 (2)	C8—C9	1.404 (3)
Mn1—N2^i^	2.2856 (17)	C8—C13	1.398 (3)
Mn1—N2	2.2855 (17)	C9—C10	1.388 (3)
Mn1—N3	2.3118 (17)	C9—C14	1.505 (3)
Mn1—N3^i^	2.3117 (17)	C10—H10	0.9300
S1—C1	1.624 (3)	C10—C11	1.387 (3)
N1—C1	1.160 (3)	C11—C12	1.382 (3)
N2—C2	1.332 (3)	C11—C15	1.514 (3)
N2—C6	1.346 (3)	C12—H12	0.9300
N3—C7	1.276 (3)	C12—C13	1.387 (3)
N3—C8	1.441 (3)	C13—C16	1.508 (3)
C2—H2	0.9300	C14—H14A	0.9600
C2—C3	1.383 (3)	C14—H14B	0.9600
C3—H3	0.9300	C14—H14C	0.9600
C3—C4	1.359 (4)	C15—H15A	0.9600
C4—H4	0.9300	C15—H15B	0.9600
C4—C5	1.384 (3)	C15—H15C	0.9600
C5—H5	0.9300	C16—H16A	0.9600
C5—C6	1.386 (3)	C16—H16B	0.9600
C6—C7	1.471 (3)	C16—H16C	0.9600
			
N1^i^—Mn1—N1	180.0	N3—C7—H7	119.0
N1—Mn1—N2	93.63 (7)	C6—C7—H7	119.0
N1—Mn1—N2^i^	86.37 (7)	C9—C8—N3	119.49 (19)
N1^i^—Mn1—N2	86.37 (7)	C13—C8—N3	119.39 (19)
N1^i^—Mn1—N2^i^	93.63 (7)	C13—C8—C9	121.1 (2)
N1—Mn1—N3^i^	91.50 (7)	C8—C9—C14	122.8 (2)
N1—Mn1—N3	88.50 (7)	C10—C9—C8	118.0 (2)
N1^i^—Mn1—N3	91.50 (7)	C10—C9—C14	119.2 (2)
N1^i^—Mn1—N3^i^	88.50 (7)	C9—C10—H10	118.8
N2—Mn1—N2^i^	180.0	C11—C10—C9	122.4 (2)
N2^i^—Mn1—N3^i^	74.26 (6)	C11—C10—H10	118.8
N2^i^—Mn1—N3	105.74 (6)	C10—C11—C15	120.3 (2)
N2—Mn1—N3	74.26 (6)	C12—C11—C10	117.7 (2)
N2—Mn1—N3^i^	105.74 (6)	C12—C11—C15	122.0 (2)
N3^i^—Mn1—N3	180.00 (7)	C11—C12—H12	118.6
C1—N1—Mn1	164.27 (19)	C11—C12—C13	122.8 (2)
C2—N2—Mn1	129.08 (15)	C13—C12—H12	118.6
C2—N2—C6	117.55 (19)	C8—C13—C16	121.9 (2)
C6—N2—Mn1	113.30 (14)	C12—C13—C8	118.0 (2)
C7—N3—Mn1	112.59 (15)	C12—C13—C16	120.1 (2)
C7—N3—C8	116.82 (19)	C9—C14—H14A	109.5
C8—N3—Mn1	129.81 (13)	C9—C14—H14B	109.5
N1—C1—S1	179.4 (2)	C9—C14—H14C	109.5
N2—C2—H2	118.5	H14A—C14—H14B	109.5
N2—C2—C3	123.1 (2)	H14A—C14—H14C	109.5
C3—C2—H2	118.5	H14B—C14—H14C	109.5
C2—C3—H3	120.5	C11—C15—H15A	109.5
C4—C3—C2	119.0 (2)	C11—C15—H15B	109.5
C4—C3—H3	120.5	C11—C15—H15C	109.5
C3—C4—H4	120.3	H15A—C15—H15B	109.5
C3—C4—C5	119.4 (2)	H15A—C15—H15C	109.5
C5—C4—H4	120.3	H15B—C15—H15C	109.5
C4—C5—H5	120.8	C13—C16—H16A	109.5
C4—C5—C6	118.4 (2)	C13—C16—H16B	109.5
C6—C5—H5	120.8	C13—C16—H16C	109.5
N2—C6—C5	122.6 (2)	H16A—C16—H16B	109.5
N2—C6—C7	117.45 (19)	H16A—C16—H16C	109.5
C5—C6—C7	119.9 (2)	H16B—C16—H16C	109.5
N3—C7—C6	122.1 (2)		
			
Mn1—N2—C2—C3	−177.25 (17)	C5—C6—C7—N3	−175.9 (2)
Mn1—N2—C6—C5	176.51 (18)	C6—N2—C2—C3	−0.8 (3)
Mn1—N2—C6—C7	−6.3 (2)	C7—N3—C8—C9	66.4 (3)
Mn1—N3—C7—C6	−3.2 (3)	C7—N3—C8—C13	−115.6 (2)
Mn1—N3—C8—C9	−102.6 (2)	C8—N3—C7—C6	−174.13 (19)
Mn1—N3—C8—C13	75.4 (2)	C8—C9—C10—C11	0.9 (4)
N2—C2—C3—C4	1.6 (4)	C9—C8—C13—C12	−1.9 (3)
N2—C6—C7—N3	6.8 (3)	C9—C8—C13—C16	−179.9 (2)
N3—C8—C9—C10	179.25 (19)	C9—C10—C11—C12	−2.3 (4)
N3—C8—C9—C14	−0.3 (3)	C9—C10—C11—C15	175.6 (2)
N3—C8—C13—C12	−179.89 (19)	C10—C11—C12—C13	1.7 (4)
N3—C8—C13—C16	2.1 (3)	C11—C12—C13—C8	0.4 (3)
C2—N2—C6—C5	−0.5 (3)	C11—C12—C13—C16	178.4 (2)
C2—N2—C6—C7	176.70 (19)	C13—C8—C9—C10	1.2 (3)
C2—C3—C4—C5	−1.0 (4)	C13—C8—C9—C14	−178.3 (2)
C3—C4—C5—C6	−0.1 (4)	C14—C9—C10—C11	−179.5 (2)
C4—C5—C6—N2	1.0 (4)	C15—C11—C12—C13	−176.3 (2)
C4—C5—C6—C7	−176.2 (2)		

Symmetry code: (i) −*x*+1, −*y*+1, −*z*+1.

### trans-Bis(thiocyanato-κN)bis{2,4,6-trimethyl-N-[(pyridin-2-yl)methylidene]aniline-κ2N,N'}manganese(II) (1) Hydrogen-bond geometry (Å, º)

Cg1 is the mid-point of the C1═N1 group. *Cg*2 and *Cg*3 are the centroids of the N2/C2–C6 and C8–C13 rings.

**Table:**

*D*—H···*A*	*D*—H	H···*A*	*D*···*A*	*D*—H···*A*
C3—H3···S1^ii^	0.93	3.16	3.812 (3)	129
C4—H4···*Cg*1^ii^	0.93	2.89	3.561 (3)	129
C5—H5···S1^iii^	0.93	3.00	3.785 (3)	143
C7—H7···S1^iii^	0.93	3.07	3.871 (2)	146
C14—H14*A*···*Cg*1^iii^	0.96	2.89	3.779 (3)	153
C14—H14*B*···*Cg*2^iii^	0.96	2.68	3.556 (3)	152
C16—H16*C*···*Cg*3^iv^	0.96	3.14	3.711 (3)	145

Symmetry codes: (ii) *x*, *y*+1, *z*; (iii) −*x*, −*y*+1, −*z*+1; (iv) −*x*+1, −*y*+1, −*z*+2.

### trans-Bis(thiocyanato-κN)bis{2,4,6-trimethyl-N-[(pyridin-2-yl)methylidene]aniline-κ2N,N'}nickel(II) (2) Crystal data

**Table:**

[Ni(NCS)_2_(C_15_H_16_N_2_)_2_]	*F*(000) = 652
*M**_r_* = 623.46	*D*_x_ = 1.333 Mg m^−^^3^
Monoclinic, *P*2_1_/*n*	Mo *K*α radiation, λ = 0.71073 Å
*a* = 9.6156 (5) Å	Cell parameters from 9899 reflections
*b* = 12.6786 (7) Å	θ = 2.9–26.0°
*c* = 12.9870 (7) Å	µ = 0.79 mm^−^^1^
β = 101.250 (2)°	*T* = 296 K
*V* = 1552.85 (14) Å^3^	Block, yellow
*Z* = 2	0.34 × 0.3 × 0.3 mm

### trans-Bis(thiocyanato-κN)bis{2,4,6-trimethyl-N-[(pyridin-2-yl)methylidene]aniline-κ2N,N'}nickel(II) (2) Data collection

**Table:**

BRUKER D8 QUEST CMOS PHOTON II diffractometer	3037 independent reflections
Radiation source: sealed x-ray tube	2486 reflections with *I* > 2σ(*I*)
Graphite monochromator	*R*_int_ = 0.032
Detector resolution: 7.39 pixels mm^-1^	θ_max_ = 26.1°, θ_min_ = 2.9°
φ and ω scans	*h* = −11→11
Absorption correction: multi-scan (*SADABS*; Krause *et al.*, 2015)	*k* = −15→15
*T*_min_ = 0.607, *T*_max_ = 0.745	*l* = −16→15
16977 measured reflections	

### trans-Bis(thiocyanato-κN)bis{2,4,6-trimethyl-N-[(pyridin-2-yl)methylidene]aniline-κ2N,N'}nickel(II) (2) Refinement

**Table:**

Refinement on *F*^2^	Primary atom site location: dual
Least-squares matrix: full	Hydrogen site location: inferred from neighbouring sites
*R*[*F*^2^ > 2σ(*F*^2^)] = 0.053	H-atom parameters constrained
*wR*(*F*^2^) = 0.104	*w* = 1/[σ^2^(*F*_o_^2^) + 2.7087*P*] where *P* = (*F*_o_^2^ + 2*F*_c_^2^)/3
*S* = 1.21	(Δ/σ)_max_ < 0.001
3037 reflections	Δρ_max_ = 0.70 e Å^−^^3^
190 parameters	Δρ_min_ = −0.46 e Å^−^^3^
0 restraints	

### trans-Bis(thiocyanato-κN)bis{2,4,6-trimethyl-N-[(pyridin-2-yl)methylidene]aniline-κ2N,N'}nickel(II) (2) Special details

**Table:**

Geometry. All esds (except the esd in the dihedral angle between two l.s. planes) are estimated using the full covariance matrix. The cell esds are taken into account individually in the estimation of esds in distances, angles and torsion angles; correlations between esds in cell parameters are only used when they are defined by crystal symmetry. An approximate (isotropic) treatment of cell esds is used for estimating esds involving l.s. planes.

### trans-Bis(thiocyanato-κN)bis{2,4,6-trimethyl-N-[(pyridin-2-yl)methylidene]aniline-κ2N,N'}nickel(II) (2) Fractional atomic coordinates and isotropic or equivalent isotropic displacement parameters (Å^2^)

**Table:**

	*x*	*y*	*z*	*U*_iso_*/*U*_eq_	
Ni1	0.500000	0.500000	0.500000	0.03174 (15)	
S1	0.30833 (12)	0.80667 (8)	0.64005 (9)	0.0656 (3)	
N1	0.4244 (3)	0.6312 (2)	0.5593 (2)	0.0407 (6)	
N2	0.7069 (3)	0.5629 (2)	0.5334 (2)	0.0376 (6)	
N3	0.5103 (3)	0.58125 (19)	0.35336 (19)	0.0351 (6)	
C1	0.3753 (3)	0.7033 (3)	0.5927 (2)	0.0380 (7)	
C2	0.8053 (4)	0.5554 (3)	0.6204 (3)	0.0533 (9)	
H2	0.783080	0.519973	0.677802	0.064*	
C3	0.9390 (4)	0.5978 (4)	0.6293 (3)	0.0729 (13)	
H3	1.005516	0.590034	0.691205	0.087*	
C4	0.9733 (4)	0.6514 (4)	0.5467 (4)	0.0819 (15)	
H4	1.062568	0.681377	0.551611	0.098*	
C5	0.8729 (4)	0.6600 (4)	0.4559 (3)	0.0678 (12)	
H5	0.893397	0.695433	0.398017	0.081*	
C6	0.7414 (3)	0.6153 (3)	0.4521 (3)	0.0437 (8)	
C7	0.6315 (3)	0.6226 (3)	0.3574 (3)	0.0444 (8)	
H7	0.650545	0.658144	0.299091	0.053*	
C8	0.4138 (3)	0.5878 (2)	0.2529 (2)	0.0372 (7)	
C9	0.3021 (4)	0.6589 (3)	0.2392 (3)	0.0462 (8)	
C10	0.2091 (4)	0.6604 (3)	0.1422 (3)	0.0545 (9)	
H10	0.134814	0.708629	0.131543	0.065*	
C11	0.2232 (4)	0.5933 (3)	0.0619 (3)	0.0517 (9)	
C12	0.3352 (4)	0.5232 (3)	0.0780 (3)	0.0538 (9)	
H12	0.345521	0.477030	0.024406	0.065*	
C13	0.4330 (4)	0.5197 (3)	0.1723 (3)	0.0451 (8)	
C14	0.2780 (5)	0.7328 (4)	0.3238 (3)	0.0732 (13)	
H14A	0.250055	0.693336	0.379517	0.110*	
H14B	0.204558	0.781995	0.295563	0.110*	
H14C	0.364035	0.770498	0.350751	0.110*	
C15	0.1202 (5)	0.5975 (4)	−0.0417 (3)	0.0734 (12)	
H15A	0.029627	0.621583	−0.030483	0.110*	
H15B	0.110203	0.528411	−0.072451	0.110*	
H15C	0.155037	0.645291	−0.088141	0.110*	
C16	0.5547 (5)	0.4432 (3)	0.1826 (3)	0.0675 (12)	
H16A	0.642709	0.480996	0.199615	0.101*	
H16B	0.550087	0.406302	0.117462	0.101*	
H16C	0.548991	0.393416	0.237354	0.101*	

### trans-Bis(thiocyanato-κN)bis{2,4,6-trimethyl-N-[(pyridin-2-yl)methylidene]aniline-κ2N,N'}nickel(II) (2) Atomic displacement parameters (Å^2^)

**Table:**

	*U*^11^	*U*^22^	*U*^33^	*U*^12^	*U*^13^	*U*^23^
Ni1	0.0322 (3)	0.0306 (3)	0.0340 (3)	−0.0017 (2)	0.0105 (2)	−0.0011 (2)
S1	0.0730 (7)	0.0543 (6)	0.0761 (7)	0.0125 (5)	0.0307 (6)	−0.0150 (5)
N1	0.0474 (15)	0.0369 (15)	0.0401 (15)	0.0012 (12)	0.0139 (12)	−0.0028 (12)
N2	0.0351 (13)	0.0347 (14)	0.0434 (15)	−0.0050 (11)	0.0087 (11)	−0.0019 (11)
N3	0.0400 (14)	0.0316 (13)	0.0355 (13)	0.0054 (11)	0.0117 (11)	0.0039 (11)
C1	0.0377 (16)	0.0437 (19)	0.0340 (16)	−0.0035 (14)	0.0105 (13)	0.0018 (14)
C2	0.048 (2)	0.062 (2)	0.047 (2)	−0.0088 (18)	0.0038 (16)	−0.0006 (18)
C3	0.046 (2)	0.102 (4)	0.064 (3)	−0.016 (2)	−0.0051 (19)	−0.004 (3)
C4	0.043 (2)	0.112 (4)	0.089 (3)	−0.030 (2)	0.008 (2)	0.005 (3)
C5	0.050 (2)	0.083 (3)	0.073 (3)	−0.023 (2)	0.017 (2)	0.011 (2)
C6	0.0392 (17)	0.0463 (19)	0.0479 (19)	−0.0060 (15)	0.0142 (15)	0.0020 (15)
C7	0.0480 (19)	0.0442 (19)	0.0441 (18)	−0.0028 (15)	0.0169 (15)	0.0078 (15)
C8	0.0398 (16)	0.0388 (17)	0.0338 (16)	0.0008 (13)	0.0093 (13)	0.0053 (13)
C9	0.0468 (19)	0.047 (2)	0.0453 (19)	0.0090 (16)	0.0105 (15)	0.0071 (16)
C10	0.049 (2)	0.057 (2)	0.057 (2)	0.0144 (17)	0.0078 (17)	0.0106 (18)
C11	0.056 (2)	0.050 (2)	0.046 (2)	−0.0018 (17)	0.0022 (16)	0.0097 (17)
C12	0.072 (2)	0.050 (2)	0.0376 (18)	0.0048 (18)	0.0062 (17)	0.0020 (15)
C13	0.056 (2)	0.0425 (19)	0.0383 (17)	0.0090 (15)	0.0121 (15)	0.0079 (14)
C14	0.085 (3)	0.073 (3)	0.059 (2)	0.033 (2)	0.008 (2)	−0.006 (2)
C15	0.073 (3)	0.078 (3)	0.061 (3)	0.002 (2)	−0.008 (2)	0.007 (2)
C16	0.089 (3)	0.072 (3)	0.043 (2)	0.036 (2)	0.016 (2)	0.0029 (19)

### trans-Bis(thiocyanato-κN)bis{2,4,6-trimethyl-N-[(pyridin-2-yl)methylidene]aniline-κ2N,N'}nickel(II) (2) Geometric parameters (Å, º)

**Table:**

Ni1—N1	2.027 (3)	C7—H7	0.9300
Ni1—N1^i^	2.027 (3)	C8—C9	1.387 (4)
Ni1—N2^i^	2.108 (2)	C8—C13	1.398 (4)
Ni1—N2	2.108 (2)	C9—C10	1.395 (5)
Ni1—N3	2.184 (2)	C9—C14	1.496 (5)
Ni1—N3^i^	2.184 (2)	C10—H10	0.9300
S1—C1	1.632 (3)	C10—C11	1.373 (5)
N1—C1	1.152 (4)	C11—C12	1.380 (5)
N2—C2	1.329 (4)	C11—C15	1.508 (5)
N2—C6	1.343 (4)	C12—H12	0.9300
N3—C7	1.269 (4)	C12—C13	1.391 (5)
N3—C8	1.448 (4)	C13—C16	1.506 (5)
C2—H2	0.9300	C14—H14A	0.9600
C2—C3	1.378 (5)	C14—H14B	0.9600
C3—H3	0.9300	C14—H14C	0.9600
C3—C4	1.363 (6)	C15—H15A	0.9600
C4—H4	0.9300	C15—H15B	0.9600
C4—C5	1.374 (6)	C15—H15C	0.9600
C5—H5	0.9300	C16—H16A	0.9600
C5—C6	1.377 (5)	C16—H16B	0.9600
C6—C7	1.459 (5)	C16—H16C	0.9600
			
N1—Ni1—N1^i^	180.00 (14)	N3—C7—H7	119.4
N1^i^—Ni1—N2	89.71 (10)	C6—C7—H7	119.4
N1^i^—Ni1—N2^i^	90.29 (10)	C9—C8—N3	119.8 (3)
N1—Ni1—N2	90.29 (10)	C9—C8—C13	121.2 (3)
N1—Ni1—N2^i^	89.71 (10)	C13—C8—N3	118.9 (3)
N1^i^—Ni1—N3^i^	91.41 (10)	C8—C9—C10	117.9 (3)
N1^i^—Ni1—N3	88.59 (10)	C8—C9—C14	122.6 (3)
N1—Ni1—N3	91.41 (10)	C10—C9—C14	119.4 (3)
N1—Ni1—N3^i^	88.59 (10)	C9—C10—H10	118.8
N2—Ni1—N2^i^	180.0	C11—C10—C9	122.5 (3)
N2^i^—Ni1—N3^i^	78.43 (10)	C11—C10—H10	118.8
N2^i^—Ni1—N3	101.57 (10)	C10—C11—C12	118.2 (3)
N2—Ni1—N3	78.43 (10)	C10—C11—C15	120.7 (4)
N2—Ni1—N3^i^	101.57 (10)	C12—C11—C15	121.1 (4)
N3^i^—Ni1—N3	180.00 (13)	C11—C12—H12	119.1
C1—N1—Ni1	176.7 (3)	C11—C12—C13	121.9 (3)
C2—N2—Ni1	129.4 (2)	C13—C12—H12	119.1
C2—N2—C6	117.4 (3)	C8—C13—C16	123.1 (3)
C6—N2—Ni1	113.2 (2)	C12—C13—C8	118.3 (3)
C7—N3—Ni1	110.9 (2)	C12—C13—C16	118.7 (3)
C7—N3—C8	115.8 (3)	C9—C14—H14A	109.5
C8—N3—Ni1	133.02 (19)	C9—C14—H14B	109.5
N1—C1—S1	179.0 (3)	C9—C14—H14C	109.5
N2—C2—H2	118.6	H14A—C14—H14B	109.5
N2—C2—C3	122.8 (4)	H14A—C14—H14C	109.5
C3—C2—H2	118.6	H14B—C14—H14C	109.5
C2—C3—H3	120.2	C11—C15—H15A	109.5
C4—C3—C2	119.6 (4)	C11—C15—H15B	109.5
C4—C3—H3	120.2	C11—C15—H15C	109.5
C3—C4—H4	120.7	H15A—C15—H15B	109.5
C3—C4—C5	118.5 (4)	H15A—C15—H15C	109.5
C5—C4—H4	120.7	H15B—C15—H15C	109.5
C4—C5—H5	120.5	C13—C16—H16A	109.5
C4—C5—C6	119.0 (4)	C13—C16—H16B	109.5
C6—C5—H5	120.5	C13—C16—H16C	109.5
N2—C6—C5	122.7 (3)	H16A—C16—H16B	109.5
N2—C6—C7	116.4 (3)	H16A—C16—H16C	109.5
C5—C6—C7	120.9 (3)	H16B—C16—H16C	109.5
N3—C7—C6	121.1 (3)		
			
Ni1—N2—C2—C3	177.4 (3)	C5—C6—C7—N3	179.8 (4)
Ni1—N2—C6—C5	−178.0 (3)	C6—N2—C2—C3	−0.6 (6)
Ni1—N2—C6—C7	1.7 (4)	C7—N3—C8—C9	−105.0 (3)
Ni1—N3—C7—C6	−1.6 (4)	C7—N3—C8—C13	76.9 (4)
Ni1—N3—C8—C9	82.2 (4)	C8—N3—C7—C6	−176.0 (3)
Ni1—N3—C8—C13	−96.0 (3)	C8—C9—C10—C11	1.2 (5)
N2—C2—C3—C4	0.9 (7)	C9—C8—C13—C12	−1.4 (5)
N2—C6—C7—N3	0.0 (5)	C9—C8—C13—C16	178.4 (3)
N3—C8—C9—C10	−178.1 (3)	C9—C10—C11—C12	−1.0 (6)
N3—C8—C9—C14	1.7 (5)	C9—C10—C11—C15	179.9 (4)
N3—C8—C13—C12	176.8 (3)	C10—C11—C12—C13	−0.4 (6)
N3—C8—C13—C16	−3.5 (5)	C11—C12—C13—C8	1.6 (5)
C2—N2—C6—C5	0.3 (5)	C11—C12—C13—C16	−178.2 (4)
C2—N2—C6—C7	−179.9 (3)	C13—C8—C9—C10	0.0 (5)
C2—C3—C4—C5	−0.9 (8)	C13—C8—C9—C14	179.8 (4)
C3—C4—C5—C6	0.6 (8)	C14—C9—C10—C11	−178.6 (4)
C4—C5—C6—N2	−0.3 (7)	C15—C11—C12—C13	178.6 (4)
C4—C5—C6—C7	179.9 (4)		

Symmetry code: (i) −*x*+1, −*y*+1, −*z*+1.

### trans-Bis(thiocyanato-κN)bis{2,4,6-trimethyl-N-[(pyridin-2-yl)methylidene]aniline-κ2N,N'}nickel(II) (2) Hydrogen-bond geometry (Å, º)

*Cg*1 is the mid-point of the C1═N1 group.

**Table:**

*D*—H···*A*	*D*—H	H···*A*	*D*···*A*	*D*—H···*A*
C4—H4···S1^ii^	0.93	2.89	3.769 (4)	158
C7—H7···S1^iii^	0.93	2.83	3.680 (3)	153
C10—H10···S1^iv^	0.93	3.17	3.871 (4)	134
C10—H10···*Cg*1^iv^	0.93	2.73	3.655 (4)	171

Symmetry codes: (ii) *x*+1, *y*, *z*; (iii) *x*+1/2, −*y*+3/2, *z*−1/2; (iv) *x*−1/2, −*y*+3/2, *z*−1/2.
